# Enhanced disease progression due to persistent HPV-16/58 infections in Korean women: a systematic review and the Korea HPV cohort study

**DOI:** 10.1186/s12985-021-01657-2

**Published:** 2021-09-17

**Authors:** Jaehyun Seong, Sangmi Ryou, JeongGyu Lee, Myeongsu Yoo, Sooyoung Hur, Byeong-Sun Choi, The Korea HPV Cohort Study

**Affiliations:** 1 Division of Clinical Research, Center for Emerging Virus Research, National Institute of Infectious Diseases, Korea National Institute of Health, Cheongju, Republic of Korea; 2 Department of Obstetrics and Gynecology, Seoul St. Mary’s Hospital, The Catholic University of Korea College of Medicine, Seoul, Republic of Korea; 3 Seoul St. Mary’s Hospital, Korea University Guro Hospital, Konkuk University Medical Center, Cha University Gangnam Medical Center, Inje University Busan Paik Hospital, Keimyung University Dongsan Medical Center, Seoul, Republic of Korea

**Keywords:** Disease progression, HPV genotype, Korea, Pap smear, Persistent infection, Systematic review

## Abstract

**Background:**

Persistent human papillomavirus (HPV) infection is a key factor for the development and progression of cervical cancer. We sought to identify the type-specific HPV prevalence by cervical cytology and assess disease progression risk based on high-risk persistent HPV infection in South Korea.

**Methods:**

To investigate the HPV prevalence by Pap results, we searched seven literature databases without any language or date restrictions until July 17, 2019. To estimate the risk of disease progression by HPV type, we used the Korea HPV Cohort study data. The search included the terms “HPV” and “Genotype” and “Korea.” Studies on Korean women, type-specific HPV distribution by cytological findings, and detailed methodological description of the detection assay were included. We assessed the risk of disease progression according to the high-risk HPV type related to the nonavalent vaccine and associated persistent infections in 686 HPV-positive women with atypical squamous cells of uncertain significance or low-grade squamous intraepithelial lesions from the Korea HPV Cohort Study. Type-specific HPV prevalence was the proportion of women positive for a specific HPV genotype among all HPV-positive women tested for that genotype in the systematic review.

**Results:**

We included 23 studies in our review. HPV-16 was the most prevalent, followed by HPV-58, -53, -70, -18, and -68. In women with high-grade squamous intraepithelial lesions, including cancer, HPV-16, -18, and -58 were the most prevalent. In the longitudinal cohort study, the adjusted hazard ratio of disease progression from atypical squamous cells of uncertain significance to high-grade squamous intraepithelial lesions was significantly higher among those with persistent HPV-58 (increase in risk: 3.54–5.84) and HPV-16 (2.64–5.04) infections.

**Conclusions:**

While HPV-16 was the most prevalent, persistent infections of HPV-16/58 increased the risk of disease progression to high-grade squamous intraepithelial lesions. Therefore, persistent infections of HPV-16 and -58 are critical risk factors for cervical disease progression in Korea. Our results suggest that equal attention should be paid to HPV-58 and -16 infections and provide important evidence to assist in planning the National Immunization Program in Korea.

**Supplementary Information:**

The online version contains supplementary material available at 10.1186/s12985-021-01657-2.

## Background

Human papillomavirus (HPV) infection is the main cause of cervical cancer. Particularly, HPV type 16 (HPV-16) and HPV-18 cause 70% of cervical cancers and pre-cancerous cervical lesions [1, 2]. Approximately 570,000 cases of cervical cancer and 311,000 deaths from the disease were reported in 2018 [3]. In Korea, the incidence of cervical cancer has been steadily decreasing from 4,443 cases in 1999 (18.9 per 100,000) to 3,500 cases in 2018 (10.5 per 100,000); however, it remains the eighth most common cancer affecting women [4].

Most human papillomavirus (HPV) infections regress spontaneously, whereas 10–15% of them may progress to precancerous lesions and then cancer with persistent HPV infections. Persistent high-risk HPV (HR-HPV) infection is strongly and consistently associated with high-grade cervical lesions and causes cervical cancer progression in more than 99.7% of women [5, 6]. According to the most updated International Agency for Research on Cancer information, the prevalence of HPV infections is 68.0% in cervical cancer (6.3% in cervical lesions with normal cytology, 33.2% in low-grade lesions, and 46.7% in high-grade lesions) among the Korean population [7, 8].

Type-specific HPV prevalence varies in different countries. Globally, HPV-16, -31, -51, and -53 are the most prevalent HPV genotypes. HPV-33 is prevalent in Europe, while HPV-52 and -58 are predominant in Asia [9, 10]. HPV-16, which is globally the most common cause of HPV-associated cancers, is also the most common type seen in Korea. Moreover, in Korea, HPV-53, -58, and -52 are the other most common HR‐HPV types detected [11, 12]. Infection by a specific type of HPV is a major risk factor in the development of cervical diseases. The prevalence of different HPV types in cervical lesions has been studied extensively.

More recently, a short-term follow-up evaluation of the Korea HPV Cohort Study showed differences in the progression risk for abnormal cervical lesions associated with specific HPV types. The rate of progression from atypical squamous cells of uncertain significance (ASCUS) to low-grade or high-grade squamous intraepithelial lesions (LSIL or HSIL) and from LSIL to HSIL was generally 15% [11]. In comparison, it was significantly higher in HPV-16-positive women than in those positive for other HPV types (27% vs. 17%) [13]. Additionally, with abnormal cervical cytology and positivity for HR-HPV types, the rate of progression to CIN3 or cancer was 7.6% [11, 13, 14].

To date, three prophylactic vaccines target various HR-HPV types, including the bivalent (HPV-16/-18), quadrivalent (HPV-6/-11/-16/-18), and the nonavalent (HPV-6/-11/-16/-18/-31/-33/-45/-52/-58) vaccines. In 2016, the bivalent and quadrivalent HPV vaccines were included as part of the National Immunization Program (NIP) to vaccinate girls aged 12 years in Korea. A significant increase in the awareness of HPV infection (35.8%) and vaccination (36.9%) was observed in 2016 from 13.3 to 8.6% in 2007 [15]. It has been already 6 years since free HPV vaccination offered by NIP in Korea. But, there is still lack of clear understanding of HPV and NIP covered HPV vaccines in the public general [16].

## Objective

This study sought to estimate the prevalence of HR-HPV and low-risk (LR)-HPV types in abnormal cervical lesions among Korean women using a systematic review of the literature. Based on the results, we analyzed the data from a 3-year follow-up to determine the disease progression in cases with persistent HR-HPV infections by genotypes related to the nonavalent vaccine. The goal was to assess the risk of progression associated with specific HR-HPV types in persistently infected women with ASCUS or LSIL using data from the Korea HPV Cohort Study from 2010 to 2019.

## Methods

### Systematic review

#### Eligibility criteria, information sources, search strategy

We followed the PRISMA guidelines for this systematic review. The study was designed using the PICO strategy as follows: P: patients who underwent cervical screening; I: intervention was HPV genotyping and cervical cytology test; C: comparator was pooled HPV type; and O: outcome was type-specific HPV prevalence based on Papanicolaou (Pap) test. We searched three international (EMBASE [Elsevier], PubMed, and Cochrane library) and four Korean (Korean Studies Information Service System, Korean Medical Database, KoreaMed [a service of the Korean Association of Medical Journal Editors], and National Digital Science Library) literature databases without any language or date restrictions. The search contained a combination of terms including “Human papillomavirus (HPV)” and “Genotype” and “Korea.” All reference lists of the articles were accessed by July 17, 2019, from the databases. Duplicate citations were removed.

#### Study selection

Studies on (1) Korean women, (2) type-specific HPV distribution by cytological findings, and (3) detailed methodological description of the detection assay were included in the analysis. Finally, full-text copies of potentially relevant papers were obtained.

#### Data extraction and assessment of the risk of bias

**Fig. 1:**
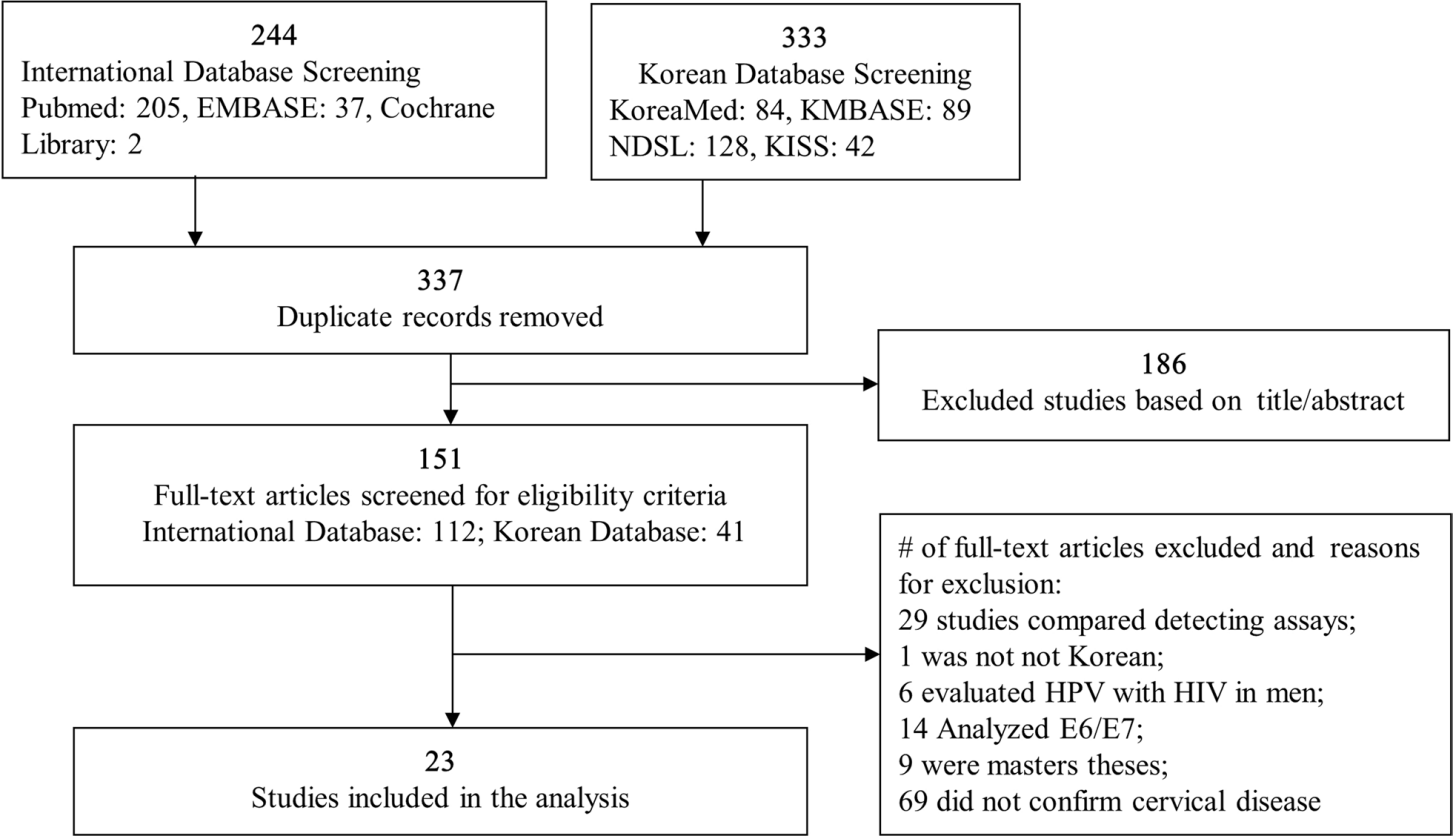
Flow diagram for the systematic review

Three independent investigators extracted data from the selected articles to minimize the risk of bias, and discrepancies were resolved by forced consensus. Based on the above criteria, two reviewers independently reviewed the full texts for data abstraction, including the following information from each study: first author, year of publication, study design, case, age, genotyping assay, detected HPV types, and cervical cytology by Pap test when available (Additional file 1: Table S1). A total of 577 citations were retrieved, and 151 full articles were reviewed. Finally, 23 studies were included in the analysis (Fig. 1).

Based on diagnosis by cytology, cervical lesions were classified into five grades: (1) normal, (2) ASCUS+ (including ASCUS, atypical glandular cells of undetermined significance; AGUS, atypical squamous cells without excluding HSIL; ASC-H), (3) LSIL, (4) HSIL, and (5) HSIL+ (including carcinoma in situ, invasive cervical cancer, squamous cell carcinoma, adenocarcinoma, adenocarcinoma in situ, and cervical cancer). HPV-16, -18, -26, -31, -33, -35, -39, -45, -51, -52, -53, -56, -58, -59, -66, -68, -72, and -82 were considered HR-HPV genotypes, while HPV-6, -11, -40, -42, -43, -44, -54, -61, -70, -72, and -81 were considered LR-HPV genotypes [2].

#### Data synthesis

Type-specific HPV prevalence was defined as the proportion of women positive for a specific HPV genotype among all HPV-positive women tested for that genotype [(number of type-specific HPV-positive women/total number of women tested) * 100]. The overall type-specific HPV prevalence was calculated using the sum of all types based on Pap results. For type-specific HPV prevalence, only studies that tested for a particular HPV type contributed to the analysis of that type; therefore, sample sizes differed between the type-specific analyses. Multiple HPV infections were separated into constituent types; thus, type-specific prevalence represents both single and multiple infections as previously defined [17].

### The Korea HPV cohort study

#### Cohort design

The Korea HPV Cohort Study has been in operation since 2010. It is a multicenter study funded by the Korea Disease Control and Prevention Agency. It was conducted at the obstetrics departments of six general hospitals across Korea to identify high-risk factors for cervical disease progression (until the stage of HSIL) among HPV-infected adult Korean women. This cohort registered participants who met the following inclusion criteria: (1) Korean women aged 20–60 years with a DNA test positive for HPV regardless of its genotype, and (2) diagnosed with ASCUS or LSIL by a Pap test. All participants provided written informed consent. All six hospitals obtained approval from their respective Institutional Review Board (IRB) to participate in the Korea HPV Cohort Study. The enrolled patients underwent HPV DNA typing and cytological examination every 6 months, and an electronic case report form was completed each time [18].

#### Analysis of disease progression to HSIL

**Fig. 2:**
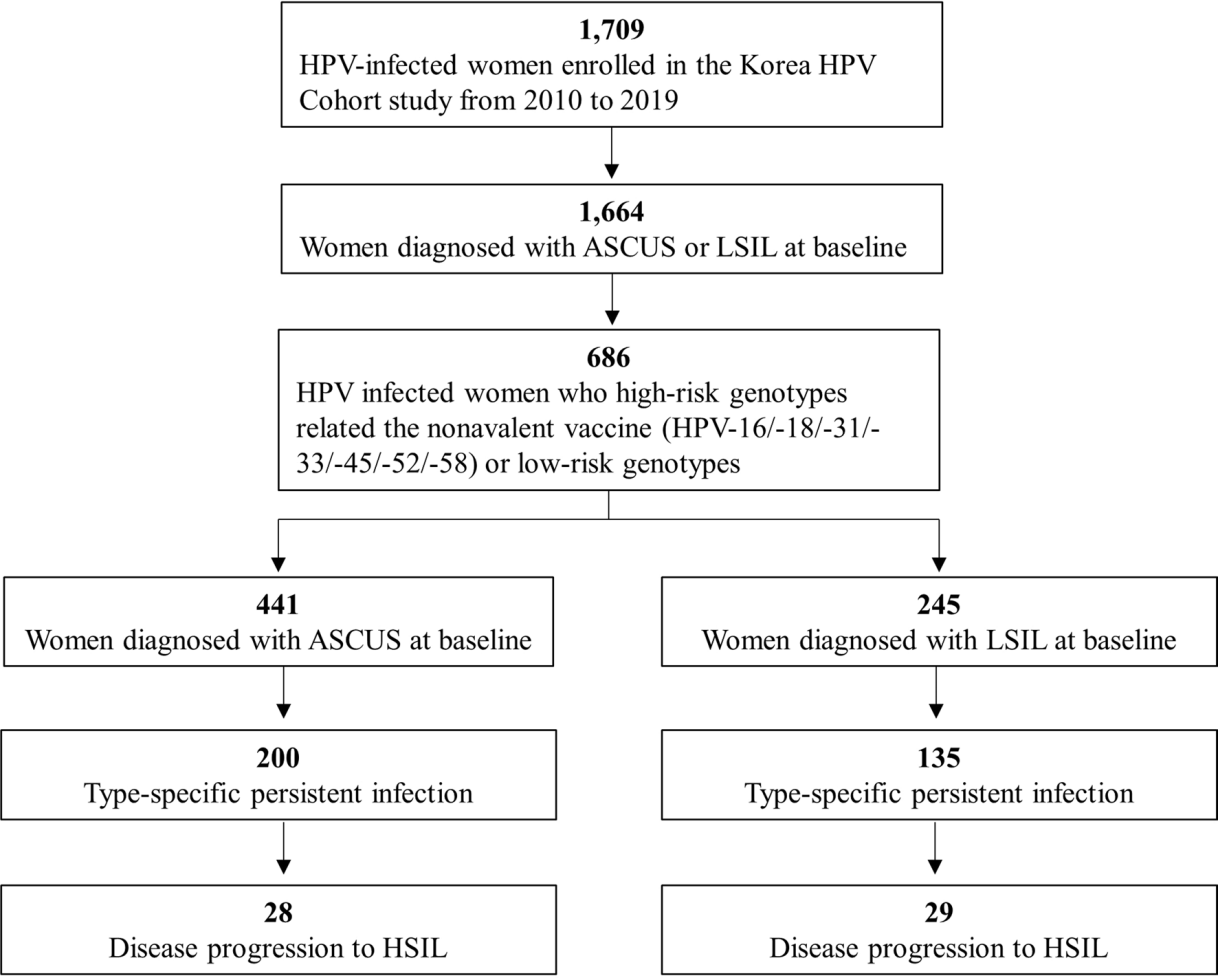
Study design to estimate the risk of disease progression with persistent infection

Between 2010 and 2019, of the 1709 registered HPV-positive women in the Korea HPV Cohort Study, 686 with (1) the HR-HPV type related to the nonavalent vaccine or (2) LR-HPV with ASCUS or LSIL were selected to be examined for the risk of disease progression to HSIL. Of the 686 selected women, 335 presented persistent infections of a genotype related to the nonavalent vaccine. Finally, we evaluated the risk of 57 women whose disease had progressed to HSIL within 36 months (Fig. 2). To confirm the potential risk of the HPV type at baseline, we examined the risk of disease progression to HSIL. Additionally, we estimated the risk of disease progression based on the type-specific HPV persistent infections. The characteristics of the women in the Korea HPV Cohort Study are shown in Additional file 2: Table S2.

We used HSIL as our primary endpoint since HSIL was the treatment threshold in the Korea HPV Cohort Study. For disease progression from ASCUS or LSIL to HSIL, all time-to-event analyses were performed from the index visit date to the date when a cytological transition to HSIL was first detected. Women who either dropped out of the study or experienced disease progression after 36 months were censored at the time of their last recorded visit. A persistent type-specific HPV infection was defined as one that was detected at two or more consecutive examinations during disease progression after HPV genotyping at baseline. Multiple HPV infections were separated into their constituent types. The risk of progression to HSIL was estimated using the Kaplan–Meier method and compared using the log-rank test. The Cox regression model was used for statistical adjustments. The hazard ratio and 95% confidence interval (95% CI) were calculated. The women’s ages and HPV genotypes were included in the multivariate model for adjustments. The analysis was conducted using SAS 9.4 (SAS Institute Inc., Cary, NC, USA) and R version 4.0.0 (www.r-project.org). A *P* value of < 0.05 was considered statistically significant. We obtained approval from the Korea Disease Control and Prevention Agency IRB for this study (approved no. 2018-06-02-P-A).

## Results

### Systematic review

#### Study selection

This systematic review included 23 studies that met the eligibility criteria. According to the cervical cytology, 17, 18, 17, 14, and 13 studies described cases with normal cytology, ASCUS+, LSIL, HSIL, and HSIL+, respectively [13, 19–40].

#### Type-specific HPV prevalence by cervical cytology

In this study, HPV-16, -58, -53, -70, -18, and -68 were the six most prevalent genotypes, and HPV-16 was the most prevalent (19.4%) among HPV-infected women. In cases with normal cytology, HPV-16 (13.8%) was the most common followed by HPV-53 (13.1%), -70 (10.6%), -58 (10.1%), and -52 (9.8%). In cases with HSIL cytology, HPV-16 was the most common type (35.3%) followed by HPV-58 (17.0%) and HPV-52 (9.0%). In cases with HSIL+ including cancer, HPV-16 was the most prevalent (54.2%), followed by HPV-18 (10.5%) and HPV-58 (7.6%). While the prevalence of HPV-68 was 9.1% in cases with normal cytology, it gradually decreased to 0.8% as the disease progressed to HSIL+ (Table 1).

**Table 1:** HPV type-specific distribution by cervical cytology

HPV type		Cervical cytology																							
		Overall^a^				Normal				ASCUS+^b^				LSIL				HSIL				HSIL+^c^			
		Study	Sample size	Positive	Prevalence (95% CI)	Study	Sample size	Positive	Prevalence (95% CI)	Study	Sample size	Positive	Prevalence (95% CI)	Study	Sample size	Positive	Prevalence (95% CI)	Study	Sample size	Positive	Prevalence (95% CI)	Study	Sample size	Positive	Prevalence (95% CI)
High-risk	HPV-16	23	16,700	3,247	19.4 (18.8–20.0)	17	8,627	1,188	13.8 (13.0–14.5)	18	4,185	635	15.2 (14.1–16.3)	17	1,293	218	16.9 (14.8–18.9)	14	1,061	375	35.3 (32.5–38.2)	13	1,534	831	54.2 (51.7–56.7)
	HPV-18	22	16,188	1,126	7.0 (6.6–7.3)	16	8,364	571	6.8 (6.3–7.4)	17	4,026	238	5.9 (5.2–6.6)	16	1,238	83	6.7 (5.3–8.1)	13	1,022	73	7.1 (5.6–8.7)	13	1,534	161	10.5 (9.0–12.0)
	HPV-26	5	9808	26	0.3 (0.2–0.4)	4	6,193	16	0.3 (0.1–0.4)	4	2,753	7	0.3 (0.1–0.4)	5	587	3	0.5 (0.0–1.1)	3	181	0	0.0 (0.0–0.0)	0	0	0	NA
	HPV-31	18	15,033	483	3.2 (2.9–3.5)	12	7,912	227	2.9 (2.5–3.2)	13	3,772	142	3.8 (3.2–4.4)	12	1,003	21	2.1 (1.2–3.0)	9	868	32	3.7 (2.4–4.9)	11	1,478	61	4.1 (3.1–5.1)
	HPV-33	19	15,138	555	3.7 (3.4–4.0)	13	7,936	222	2.8 (2.4–3.2)	14	3,795	142	3.7 (3.1–4.3)	13	1,040	38	3.7 (2.5–4.8)	10	889	67	7.5 (5.8–9.3)	11	1,478	86	5.8 (4.6–7.0)
	HPV-35	20	15,687	585	3.7 (3.4–4.0)	14	8,204	308	3.8 (3.3–4.2)	15	3,886	152	3.9 (3.3–4.5)	14	1,106	35	3.2 (2.1–4.2)	11	968	52	5.4 (4.0–6.8)	12	1,523	38	2.5 (1.7–3.3)
	HPV-39	19	14,455	647	4.5 (4.1–4.8)	13	7,523	442	5.9 (5.3–6.4)	14	3,661	134	3.7 (3.1–4.3)	13	997	46	4.6 (3.3–5.9)	10	837	11	1.3 (0.5–2.1)	11	1,437	14	1.0 (0.5–1.5)
	HPV-45	15	13,906	263	1.9 (1.7–2.1)	11	7,579	152	2.0 (1.7–2.3)	11	3,248	52	1.6 (1.2–2.0)	11	854	14	1.6 (0.8–2.5)	8	835	11	1.3 (0.5–2.1)	10	1,390	34	2.4 (1.6–3.3)
	HPV-51	18	15,062	788	5.2 (4.9–5.6)	13	7,792	441	5.7 (5.1–6.2)	14	3,856	204	5.3 (4.6–6.0)	14	1,140	104	9.1 (7.5–10.8)	11	901	34	3.8 (2.5–5.0)	10	1,349	5	0.4 (0.0–0.7)
	HPV-52	23	16,700	1,593	9.5 (9.1–10.0)	17	8,627	842	9.8 (9.1–10.4)	18	4,185	493	11.8 (10.8–12.8)	17	1,293	113	8.7 (7.2–10.3)	14	1,061	95	9.0 (7.2–10.7)	13	1,534	50	3.3 (2.4–4.1)
	HPV-53	14	13,781	1,474	10.7 (10.2–11.2)	10	7,312	956	13.1 (12.3–13.8)	12	3,633	362	10.0 (9.0–10.9)	11	964	115	11.9 (9.9–14.0)	8	699	26	3.7 (2.3–5.1)	8	1,173	15	1.3 (0.6–1.9)
	HPV-56	21	15,323	845	5.5 (5.2–5.9)	15	7,870	465	5.9 (5.4–6.4)	16	3,870	215	5.6 (4.8–6.3)	15	1,154	119	10.3 (8.6–12.1)	12	947	23	2.4 (1.4–3.4)	12	1,482	23	1.6 (0.9–2.2)
	HPV-58	23	16,700	1,790	10.7 (10.2–11.2)	17	8,627	875	10.1 (9.5–10.8)	18	4,185	477	11.4 (10.4–12.4)	17	1,293	142	11.0 (9.3–12.7)	14	1,061	180	17.0 (14.7–19.2)	13	1,534	116	7.6 (6.2–8.9)
	HPV-59	16	13,602	266	2.0 (1.7–2.2)	11	6,873	159	2.3 (2.0–2.7)	13	3,635	75	2.1 (1.6–2.5)	12	956	13	1.4 (0.6–2.1)	9	814	5	0.6 (0.1–1.2)	8	1,324	14	1.1 (0.5–1.6)
	HPV-66	16	13,394	575	4.3 (3.9–4.6)	10	6,795	330	4.9 (4.3–5.4)	13	3,635	152	4.2 (3.5–4.8)	11	881	62	7.0 (5.3–8.7)	8	750	14	1.9 (0.9–2.8)	8	1,333	17	1.3 (0.7–1.9)
	HPV-68	16	13,615	900	6.6 (6.2–7.0)	11	6,873	623	9.1 (8.4–9.7)	11	3,537	209	5.9 (5.1–6.7)	12	956	46	4.8 (3.5–6.2)	9	814	11	1.4 (0.6–2.1)	9	1,412	11	0.8 (0.3–1.2)
	HPV-73	3	9,340	55	0.6 (0.4–0.7)	2	5,836	37	0.6 (0.4–0.8)	2	2,281	15	0.7 (0.3–1.0)	2	390	3	0.8 (0.0–1.6)	1	127	0	0.0 (0.0–0.0)	1	706	0	0.0 (0.0–0.0)
	HPV-82	4	9,887	159	1.6 (1.4–1.9)	4	6,178	104	1.7 (1.4–2.0)	4	2,860	44	1.5 (1.1–2.0)	4	601	7	1.2 (0.3–2.0)	3	233	4	1.7 (0.0–3.4)	1	15	0	0.0 (0.0–0.0)
Low-risk	HPV-6	8	3,319	65	2.0 (1.5–2.4)	6	926	34	3.7 (2.5–4.9)	7	948	15	1.6 (0.8–2.4)	7	392	7	1.8 (0.5–3.1)	5	529	3	0.6 (0.0–1.2)	5	524	6	1.1 (0.2–2.1)
	HPV-11	9	4,260	44	1.0 (0.7–1.3)	6	896	11	1.2 (0.5–1.9)	6	964	9	0.9 (0.3–1.5)	7	490	9	1.8 (0.6–3.0)	6	608	10	1.6 (0.6–2.7)	6	1,302	5	0.4 (0.0–0.7)
	HPV-40	9	3,543	34	1.0 (0.6–1.3)	7	840	14	1.7 (0.8–2.5)	6	1,010	6	0.6 (0.1–1.1)	7	317	0	0.0 (0.0–0.0)	6	620	8	1.3 (0.4–2.2)	6	603	6	1.0 (0.2–1.8)
	HPV-42	6	2,796	19	0.7 (0.4–1.0)	6	886	6	0.7 (0.1–1.2)	6	757	6	0.8 (0.2–1.4)	6	402	5	1.2 (0.2–2.3)	4	295	2	0.7 (0.0–1.6)	5	456	0	0.0 (0.0–0.0)
	HPV-43	8	3,153	27	0.9 (0.5–1.2)	8	1,013	11	1.1 (0.4–1.7)	7	760	12	1.6 (0.7–2.5)	8	480	2	0.4 (0.2–1.0)	6	365	2	0.5 (0.0–1.3)	6	535	0	0.0 (0.0–0.0)
	HPV-44	8	3,152	14	0.4 (0.2–0.7)	6	631	3	0.5 (0.1–1.0)	6	626	3	0.5 (0.0–1.0)	6	328	1	0.3 (0.0–0.9)	4	326	3	0.9 (0.0–2.0)	7	1,241	4	0.3 (0.0–0.6)
	HPV-54	7	2,928	75	2.6 (2.0–3.1)	4	704	33	4.7 (3.1–6.2)	6	1,079	31	2.9 (1.9–3.9)	5	349	1	0.3 (0.0–0.8)	3	407	2	0.5 (0.0–1.2)	4	389	8	2.1 (0.6–3.5)
	HPV-61	2	1,069	29	2.7 (1.7–3.7)	2	357	13	3.6 (1.7–5.6)	2	472	12	2.5 (1.1–4.0)	2	186	4	2.2 (0.1–4.2)	2	54	0	0.0 (0.0–0.0)	0	0	0	NA
	HPV-70	10	9,827	768	7.8 (7.3–8.3)	7	4,200	447	10.6 (9.7–11.6)	8	3,221	258	8.0 (7.1–8.9)	8	672	43	6.4 (4.5–8.2)	6	601	15	2.5 (1.2–3.7)	6	1,110	5	0.5 (0.1–0.8)
	HPV-72	3	755	8	1.1 (0.3–1.8)	3	206	5	2.4 (0.3–4.5)	3	217	2	0.9 (0.0–2.2)	3	162	1	0.6 (0.0–1.8)	3	140	0	0.0 (0.0–0.0)	1	15	0	0.0 (0.0–0.0)
	HPV-81	4	1,719	24	1.4 (0.8–2.0)	4	598	11	1.8 (0.8–2.9)	4	647	9	1.4 (0.5–2.3)	4	285	4	1.4 (0.0–2.8)	3	152	0	0.0 (0.0–0.0)	3	37	0	0.0 (0.0–0.0)
	Other	14	12,864	216	1.7 (1.5–1.9)	10	6,790	81	1.2 (0.9–1.5)	10	3,239	74	2.3 (1.8–2.8)	11	907	34	3.7 (2.5–5.0)	8	513	3	0.6 (0.0–1.2)	8	1,329	24	1.8 (1.1–2.5)

ASCUS: Atypical squamous cells of uncertain significance; AGUS: Atypical glandular cells of undetermined significance; ASC-H: Atypical squamous cells without excluding HSIL; LSIL: Low-grade squamous intraepithelial lesion; HSIL: High-grade squamous intraepithelial lesion; CIS: Carcinoma in situ; ICC: Invasive cervical cancer; SCC: Squamous cell carcinoma; AC: Adenocarcinoma; AIS: Adenocarcinoma in situ; 95% CI: 95% Confidence interval

^a^Overall prevalence by cytology was calculated using the sum of all types obtained from the Pap results

^b^ASCUS including AGUS and ASC-H

^c^HSIL including CIS, ICC, SCC, AC, AIS, and cervical cancer

### The Korea HPV cohort study

#### Risk of disease progression by HPV type

During the follow-up period, the disease progressed to HSIL in 105 out of 686 women. The probability of ASCUS progression (at baseline) to HSIL within 36 months was 17.6% (60 out of 441 women; 95% CI = 13.3–21.7). In the type-specific analysis, after adjusting for age and genotype, using the LR-HPV-infected women as the control group, the aHR (adjusted hazard ratio) for disease progression to HSIL was significantly higher for HPV-16 (aHR = 2.64; 95% CI = 1.18–5.90), HPV-31 (aHR = 3.96; 1.43–10.94), and HPV-58 (aHR = 3.54; 1.66–7.55). Depending on the type-specific HPV persistent infection, the probability of disease progression increased from 17.6 to 30.7%. The highest risk of disease progression was seen with persistent HPV-58 infection (aHR = 5.84; 1.83–18.67), followed by persistent HPV-16 infection (aHR = 5.04; 1.40–18.11) (Table 2).

**Table 2:** Disease progression from ASCUS to HSIL by HPV types

	N	Progression	Probability of progression within 36 months (95% CI)^a^	Hazard ratio (95% CI)		*P* value^c^
				Unadjusted	Adjusted^b^	
*ASCUS to HSIL*	441	60	17.6 (13.3–21.7)			
Age						
20–29	76	7	13.9 (3.4–23.4)	Ref	Ref	
30–39	142	21	18.4 (10.8–25.5)	1.64 (0.70–3.87)	1.51 (0.64–3.56)	0.351
40–49	128	20	19.7 (11.4–27.3)	1.65 (0.70–3.91)	1.67 (0.70–4.00)	0.245
50–59	95	12	15.9 (6.7–24.1)	1.32 (0.52–3.36)	1.21 (0.47–3.10)	0.699
HPV type at baseline						
Low-risk	155	10	8.6 (3.1–13.8)	Ref	Ref	
HPV-16	93	15	19.7 (9.9–28.4)	2.67 (1.12–5.94)	2.64 (1.18–5.90)	0.018
HPV-18	44	3	10.6 (0.0–21.5)	1.15 (0.32–4.17)	1.08 (0.29–3.97)	0.913
HPV-31	24	6	30.9 (5.8–49.3)	4.01 (1.46–11.04)	3.96 (1.43–10.94)	0.008
HPV-33	18	3	20.6 (0.0–39.0)	2.42 (0.67–8.81)	2.34 (0.64–8.54)	0.198
HPV-45	13	2	20.5 (0.0–42.3)	2.24 (0.49–10.21)	2.16 (0.47–9.90)	0.320
HPV-52	87	12	19.6 (8.2–29.5)	2.37 (1.02–5.48)	2.32 (1.00–5.42)	0.051
HPV-58	89	21	28.5 (16.9–38.4)	3.64 (1.71–7.73)	3.54 (1.66–7.55)	0.001
Type-specific persistent infection	200	28	30.7 (14.6–43.7)			
Low-risk	88	4	6.6 (0.0–12.9)	Ref	Ref	
HPV-16	27	6	29.2 (5.2–47.1)	4.86 (1.37–17.23)	5.04 (1.40–18.11)	0.013
HPV-18	10	2	28.9 (0.0–56.7)	4.13 (0.76–22.65)	4.59 (0.82–25.70)	0.083
HPV-31	8	2	31.4 (0.0–59.7)	4.12 (0.75–22.56)	3.86 (0.70–21.39)	0.122
HPV-33	6	1	33.3 (0.0–70.0)	2.58 (0.29–23.46)	3.01 (0.33–27.56)	0.331
HPV-45	4	1	NA	3.01 (0.34–27.02)	2.80 (0.31–25.27)	0.359
HPV-52	34	4	13.8 (0.0–27.6)	2.43 (0.61–9.74)	2.57 (0.64–10.39)	0.185
HPV-58	33	10	61.7 (6.1–84.4)	5.46 (1.71–17.42)	5.84 (1.83–18.67)	0.003

ASCUS: Atypical squamous cells of uncertain significance; LSIL: Low-grade squamous intraepithelial lesion; HSIL: High-grade squamous intraepithelial lesion; 95% CI: 95% confidence interval

^a^Probabilities of progression were estimated by Kaplan–Meier survival analysis

^b^Hazard ratios and 95% confidence intervals were adjusted by age and genotype

^c^*P* values were calculated in the adjusted analysis

In women with LSIL at baseline (n = 245), the probability of progression to HSIL within 36 months was 25.7% (95% CI = 18.3–32.3). Compared to the LR-HPV group, the risk of disease progression was highest for HPV-16 (aHR = 4.71; 2.02–11.02), followed by HPV-58 (aHR = 4.37; 1.84–10.41), HPV-33 (aHR = 4.17; 1.25–13.94), and HPV-52 (aHR = 3.42; 1.23–9.52). In women with LSIL and type-specific persistent infections, the probability of disease progression increased to 38.8%. Using the LR-HPV persistently infected women as the control group, those with HPV-33 (aHR = 4.56; 1.16–18.04), -16 (aHR = 4.24; 1.49–12.10), and -58 (aHR = 4.18; 1.54–11.33) were found to be at a significantly higher risk of disease progression (Table 3). We found no association between the age or type of infection (single/multiple) and disease progression in this study.

**Table 3:** Disease progression from LSIL to HSIL by HPV types

	N	Progression	Probability of progression within 36 months (95% CI)^a^	Hazard ratio (95% CI)		*P* value^c^
				Unadjusted	Adjusted^b^	
*LSIL to HSIL*	245	45	25.7 (18.3–32.3)			
Age						
20–29	59	10	22.5 (8.4–34.3)	Ref	Ref	
30–39	71	17	30.6 (16.3–42.5)	1.30 (0.60–2.84)	1.30 (0.58–2.90)	0.520
40–49	69	15	32.0 (15.8–45.0)	1.14 (0.51–2.53)	1.10 (0.49–2.48)	0.818
50–59	46	3	10.5 (0.0–21.2)	0.34 (0.09–1.24)	0.39 (0.11–1.48)	0.167
HPV type at baseline						
Low-risk	104	8	10.9 (2.9–18.4)	Ref	Ref	
HPV-16	49	17	45.2 (24.8–60.0)	4.69 (2.03–10.88)	4.71 (2.02–11.02)	0.001
HPV-18	22	4	20.9 (0.3–37.3)	2.34 (0.71–7.78)	2.72 (0.81–9.13)	0.105
HPV-31	12	2	16.7 (0.0–35.3)	3.08 (0.65–14.53)	2.87 (0.60–13.68)	0.186
HPV-33	14	4	68.2 (0.0–92.8)	3.96 (1.19–13.18)	4.17 (1.25–13.94)	0.021
HPV-45	9	1	11.1 (0.0–29.4)	1.59 (0.20–12.74)	2.96 (0.36–24.64)	0.316
HPV-52	34	7	22.2 (6.1–35.5)	2.64 (0.96–7.29)	3.42 (1.23–9.52)	0.019
HPV-58	51	15	43.1 (22.0–58.5)	4.17 (1.77–9.84)	4.37 (1.84–10.41)	0.001
Type-specific persistent infection	135	29	38.8 (22.4–51.7)			
Low-risk	78	7	12.4 (2.8–21.0)	Ref	Ref	
HPV-16	15	8	66.6 (20.5–85.9)	4.01 (1.45–11.10)	4.24 (1.49–12.10)	0.007
HPV-18	5	1	20.0 (0.0–48.4)	2.04 (0.25–16.62)	2.25 (0.27–18.44)	0.451
HPV-31	2	0	NA	NA	NA	NA
HPV-33	5	3	70.0 (0.0–93.7)	5.95 (1.53–23.09)	4.56 (1.165–18.04)	0.030
HPV-45	1	0	NA	NA	NA	NA
HPV-52	12	2	9.1 (0.0–24.6)	1.49 (0.31–7.18)	1.81 (0.37–8.91)	0.464
HPV-58	20	9	50.3 (17.0–70.3)	4.23 (1.58–11.37)	4.18 (1.54–11.33)	0.005

ASCUS: Atypical squamous cells of uncertain significance; LSIL: Low-grade squamous intraepithelial lesion; HSIL: High-grade squamous intraepithelial lesion; 95% CI: 95% confidence interval

^a^Probabilities of progression were estimated by Kaplan–Meier survival analysis

^b^Hazard ratios and 95% confidence intervals were adjusted by age and genotype

^c^P-values were calculated in the adjusted analysis

As shown in Tables 2 and 3, in 182 women with persistent HR-HPV infections, who were characterized by ASCUS or LSIL cytology, HPV-16 (n = 42, 23.1% of HR persistent infection), -52 (n = 46, 25.3% of HR persistent infection), and -58 (n = 53, 29.1% of HR persistent infection) were found to be the most prevalent HR-HPV strains. Furthermore, the estimated progression time (to HSIL) was 19 months for 49 lesions. The median time needed for progression was 18.9, 17.9, and 12.4 months for HPV-16, -18, and -52, respectively. HR-HPVs related to HPV-16, including HPV-31, -33, and -58, required approximately 20 months. HPV-58 was an HR genotype with a high probability of progressing to HSIL within 36 months in cases with ASCUS (61.7%) and LSIL (50.3%).

**Fig. 3:**
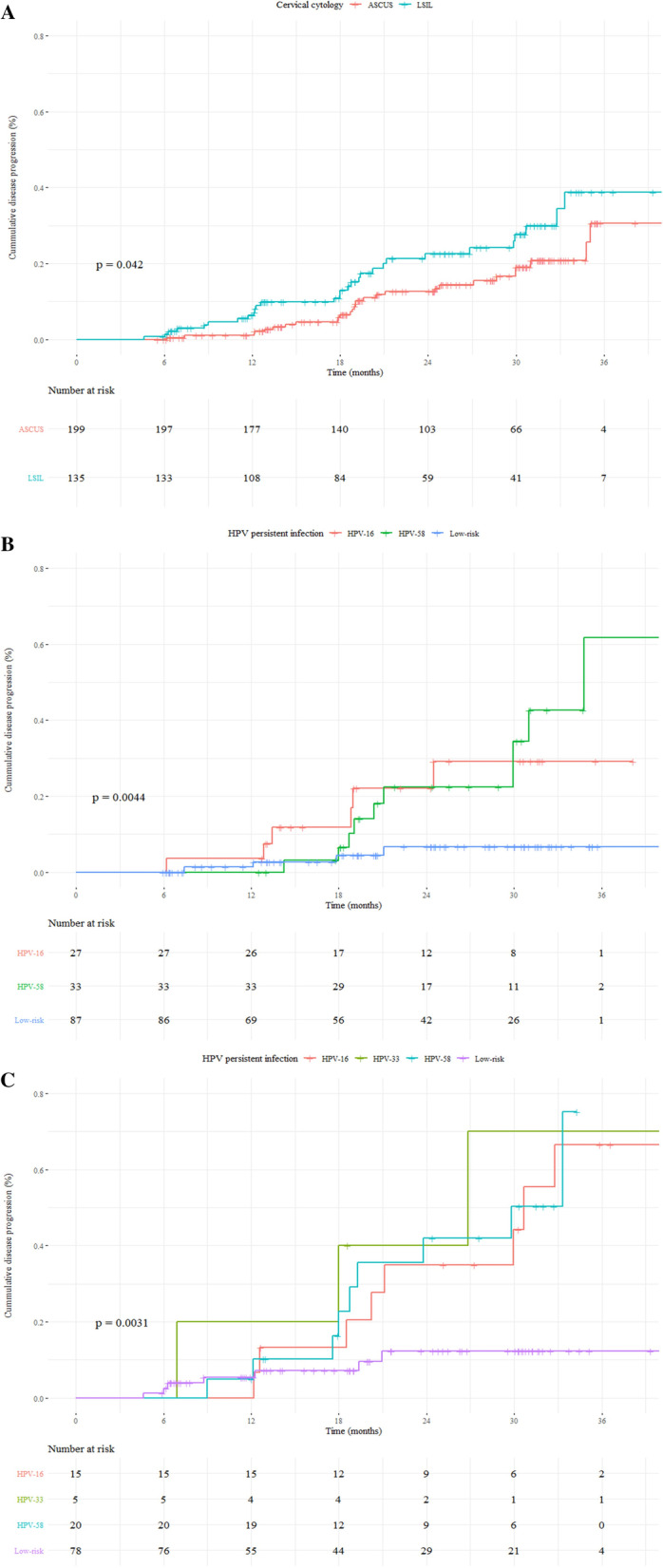
Cumulative risk of disease progression to HSIL within 36 months. Shown is the cumulative risk of disease progression **a** overall, **b** from ASCUS to HSIL by HPV genotype, and **c** from LSIL to HSIL by HPV genotype. A Kaplan–Meier plot was used to estimate the cumulative risk of disease progression to HSIL within 36 months. Shown is the cumulative risk of disease progression **a** by cervical cytology at baseline, **b** from ASCUS to HSIL by HPV persistent infection, and **c** from LSIL to HSIL by HPV persistent infection. Results in Fig. 3b and c represent only significant HPV genotypes in the multivariate analysis. *P* value was calculated by a log-rank test. ASCUS: Atypical squamous cells of uncertain significance; HSIL: High- grade squamous intraepithelial lesion; LSIL: Low-grade squamous intraepithelial lesion; HPV: human papillomavirus

From data on persistent HPV infections, we graphed the cumulative risk of disease progression to HSIL during 36 months of follow-up after the baseline infection using the Kaplan–Meier survival method. The cumulative probability of progression to HSIL from ASCUS or LSIL was 30.7% (95% CI = 14.6–43.7) and 38.8% (22.4–51.7), respectively. The survival curve was significantly different depending on the baseline cytology (log-rank test; *P* = 0.042) (Fig. 3a). We also analyzed the cumulative risk of disease progression according to the type-specific persistent infection based on baseline cytology. In women with ASCUS at baseline, the cumulative risk of HPV-58, -16, and LR-HPV was 61.7% (6.1–84.4), 29.2% (5.2–47.1), and 6.6% (0.0–12.9), respectively (log-rank test; *P* = 0.0044) (Fig. 3b). In contrast, in women with LSIL at baseline, the disease progression risks for HPV-16, -33- 52, and LR-HPV were 66.6% (20.5–85.9), 70.0% (0.0–93.7), and 50.3% (17.0–70.3), respectively (Fig. 3c).

## Discussion

### Main findings

We first performed a systematic review to analyze the prevalence of clinically relevant HPV types in Korea. We also determined the progression risk based on type-specific persistent infections in women with ASCUS or LSIL using data from a prospective cohort study in Korea. We found that HPV-16, -58, -53, -52, and -18 were the prevalent HPV types based on clinical cervical cytology. HPV-16 was the dominant genotype, with an overall prevalence of 19.4%. It was the most predominant type in patients with HSIL and worse (HSIL+). Although the prevalence of HPV-18 was lower than that of HPV-53 and HPV-58, it was dominant in HSIL+ patients. In cases with HSIL+, the most prevalent genotypes were HPV-16, -18, and -58. In this review, which was conducted using a systematic analysis of the prevalence of HPV types in cervical lesions, HPV-58, which is included in the nonavalent vaccine, was frequently found in lesions with normal cytology (10.1%), ASCUS (11.4%), LSIL (11.0%), HSIL (17.0%), and HSIL+ (7.6%). Thus, more attention should be paid to HPV-50s infections, especially HPV-58.

Furthermore, based on the 3-year observational cohort, we found that while the risk of persistent infection by HPV-16 was 5.04, it was 5.84 by HPV-58, suggesting that in addition to HPV-16 and HPV-18, vaccines against HPV-52 and HPV-58 will also be beneficial for Korean women.

### Strengths and limitations

We investigated the HPV prevalence by Pap results and the risk of disease progression by persistent HPV types. Hence, we used two methodologies: a systematic review and a prospective cohort analysis. Our study's major strength is that it combines and summarizes the results of independent case–control studies and a 3-year follow-up cohort.

Our systematic review has some limitations. First, as this study included HPV-positive Korean women, the results cannot be generalized to other populations. Second, due to the non-standardized HPV typing and Pap testing methods used across the studies, the results might be biased. Third, we did not conduct meta-analysis because of the differences in study setting, participants, and detection assay methods.

There were also several limitations in the longitudinal cohort study. First, because the Korea HPV Cohort Study aimed to investigate HPV infections in women with ASCUS or LSIL, we could not include HPV-negative women or women with normal cytology as a control group. Therefore, assessing the risk of progression of persistent LR-HPV infections was not possible. Second, since women diagnosed with CIN II+ or HSIL were treated by conization during the follow-up period of the Korea HPV Cohort Study, we could not include women with HSIL+. Finally, our study lacked a sufficient sample size. Had the study population been larger, we could have potentially revealed a more significant risk of disease progression by HPV genotype.

### Comparison with existing literature

In the systematic review, we found that HPV-16, -58, -53, -52, and -18 were the prevalent HPV types based on clinical cervical cytology. It was known that the distribution of HPV type varies by region. HPV-16, -52, and -56 were prevalent in European region, HPV-53, -62, and -58 were prevalent in South American region, and HPV-16, -58, and -82 were prevalent in Eastern Mediterranean region [41]. Compared with other Asian countries, Korea has a higher prevalence of HPV-53 and -58 and incidence of cervical cancer associated with HPV-52 and -58 [42]. Although, HPV-45 which is most closely related to HPV-18, is an HR oncogenic genotype in sub-Saharan Africa [10], it seems to be less influenced in the Korean population (1.9%). HPV-66 and -68 showed a higher prevalence in normal and low-grade cervical lesions, decreasing with disease progression. These results indicate that a cervix disease is closely related to the HPV type, and its genotype distribution differs regionally.

In our prospective study, the highest risk for disease progression to HSIL was noted with persistent HPV-58 and HPV-16 infections. In the clinical trial, risk of disease progression associated with persistent infection of the same type was significantly higher for oncogenic types than for non-oncogenic types. Hazard ratios for progression of CIN2+ to HPV-16, -33, 31, 45, and -18 were 10.4, 9.6, 5.6, 5.3, and 3.8, respectively [43].

Some studies point to an association between multiple types of HPV and persistent infections [42, 44, 45]. Compared to a single infection, multiple HR-HPV infections place an individual at a higher risk of developing cervical cancer. However, there is no general agreement on this. Consistent with the results of previous studies [46], we found no significant increase in the risk of disease progression with multiple persistent infections (*P* = 0.986) (Additional file 3: Table S3). We also found that age had no significant effect on the risk of disease progression.

## Conclusions

We assessed the association between disease progression and persistent HR-HPV infection with the nonavalent vaccine-related genotypes. Particularly, we found that the risk of disease progression from ASCUS to HSIL was higher with persistent HPV-58 and HPV-16 infections. Therefore, equal attention should be paid to these two genotypes. However, regardless of persistent infections, patients going from LSIL to HSIL did not show differences in disease progression risk. Because ASCUS was regarded as the relatively initial stage based on cytology, it appears that persistent infections of HR-HPV types can affect disease progression from ASCUS to HSIL. We believe that our findings will provide scientific data to assist in detailed planning for the HPV NIP. Further studies with a larger sample size and longer follow-up periods are required to understand better the role of HPV and vaccination in cancer development.

In 2016, Korea initiated bivalent and quadrivalent vaccines as a part of the NIP to vaccinate girls aged 12 years. Both the vaccines are preventive against the HPV-16/18 genotypes that cause 70% of cervical cancers. Knowing the prevalence and genotypes of HPV in a vaccinated population is critical for monitoring the vaccines' efficacy; however, there is no official data on the HPV vaccination in Korean adult women. We recently reported the status of HPV vaccination and its effectiveness in HPV-infected women aged 20–60 years with an HPV vaccine history in Korea. In 1300 women, the prevalence ratio of HPV-16/18 in the 335 vaccinated women (greater than 12 months beforehand) was 0.51 (95% CI = 0.29–0.88) compared with that in the unvaccinated women, which was significant, thereby indicating the positive impact of vaccination [47].

In conclusion, persistent infection of not only HPV-16 but also HPV-58 is a critical risk factor for the progression of cervical disease in Korea. Persistent infections by HR-HPV genotypes, including those in the nonavalent vaccine, provide clues regarding disease progression to HSIL/HSIL+. In this 36-month longitudinal study, we found that the risk of progression in patients with persistent infections varied considerably depending on HR genotypes. We believe that our findings will provide scientific data to assist the detailed planning for the National Immunization Program for HPV.

## Supplementary Information

**Additional file 1**. Characteristics of the studies included in the systematic review.

**Additional file 2**. Basic characteristics of women at baseline in the Korea HPV Cohort Study (n = 1,664).

**Additional file 3**. Risk of disease progression to HSIL by persistent HPV infections (single/multi-infection).

## Data Availability

The datasets supporting the conclusions of this article are included within the article and its additional file.

## Abbreviations

HPV: Human papilloma virus
HR-HPV: High-risk HPV
LR-HPV: Low-risk HPV
ASCUS: Atypical squamous cells of uncertain significance
LSIL: Low-grade squamous intraepithelial lesions
HSIL: High-grade SIL
NIP: National immunization program
IRB: Institutional review board
CI: Confidence interval
aHR: adjusted Hazards ratio
pap: Papanicolaou
