# Supplementary material for: Enhanced disease progression due to persistent HPV-16/58 infections in Korean women: a systematic review and the Korea HPV cohort study
Source: Virol J. 2021 Sep 17;18:188. doi: 10.1186/s12985-021-01657-2 (PMC8447749; doi:10.1186/s12985-021-01657-2)
Supplement: Supplementary file 1 — Additional file 1. Characteristics of the studies included in the systematic review. [file 12985_2021_1657_MOESM1_ESM.docx]

**Additional Table 1. Characteristics of the studies included in the systematic review.**

| **Author, year [Reference]** | **Database** | **Study design** | **Study population** | **Case (n)*** | **Age** | **Detection and genotyping assay** | **Detected HPV types** | **Cervical cytology** |
| --- | --- | --- | --- | --- | --- | --- | --- | --- |
| Kahng and Lee, 2008^25^ | EMBASE | Case control study | 1211 | 319 | 20-70 | HPV DNA Chip kit (BioMedLab) | 6, 11, 16, 18, 31, 33, 34, 35, 39, 40, 42, 43, 44, 45, 51, 52, 56, 58, 59, 66, 68, and 69 | Normal, ASCUS, LSIL, and HSIL |
| Nah et al., 2017^30^ | EMBASE | Cross-sectional study | 18815 | 5802 | 20-99(median; 53) | Anyplex II HPV 28 (Seegene) | 6, 11, 16, 18, 26, 31, 33, 35, 39, 40, 42, 43, 44, 45, 51, 52, 53, 54, 56, 58, 59, 61, 66, 68, 69, 70, 73, and 82 | Normal, ASCUS, LSIL, HSIL, SCC, AC |
| Oh et al., 2010^31^ | EMBASE | Case control study | 874 | 706 | 39-60 | SPF10 PCR DEIA and LiPA25 assay  (Labo Biomedical) | 6, 11, 16, 18, 31, 33, 35, 39, 45, 52, 56, 58, 59, 68, and 73 | ICC |
| Shin et al., 2003^34^ | Cochran library | Case control study | 863 | 105 | 20-74(Median; 44) | DNA polymerase chain reaction-  enzyme immunoassay (PCR-EIA) | 6, 16, 18, 31, 33, 35, 39, 40, 42, 43, 44, 45, 51, 52, 53, 54, 56, 58, 59, 66, 68, 70, 81, 84, and CP6108 | Normal, ASCUS, LSIL, HSIL, CIS, and ICC |
| Hong et al., 2009^36^ | EMBASE | Case control study | 2368 | 744 | 20-94(mean; 45.7) | My HPV DNA chip (Mygene) | 6, 11, 16, 18, 31, 33, 34, 35, 39, 40, 42, 43, 44, 45, 51, 52, 53, 54, 56, 58, 59, 66, 68, and 70 | Normal, ASCUS, ASCH/AGC, LSIL, HSIL, AIS, and IC |
| Kim et al., 2013^26^ | KISS | Cross-sectional study | 7014 | 583 | 20-60(mean 48.2±9.2 | HPV DNA chip kit  (Biomed lab) | 6, 11, 16, 18, 31, 33,34, 35, 39, 40, 42, 43, 44, 45, 51, 52, 54,56, 58, and 59 | Normal, , HSIL, and SCCA |
| Lee et al., 2009^27^ | KISS | Case control study | 2562 | 964 | 20-73(median; 37.6) | HPV L1 gene PCR-BLAST SEARCH | 6, 11, 16, 18, 22, 26, 30, 31, 32, 33, 34, 35, 39, 40, 42, 43, 44, 45, 51, 52, 53, 54,56, 58, 59, 61, 62, 66, 68, 72,73, 81, and 82 | Normal, ASCUS, LSIL, and HSIL |
| Kim et al., 2012^37^ | KISS | Case control study | 704 | 865 | 20-59 | MolecuTech REBA HPV-ID® Kit  (YD Diagnostics) | 6, 11, 16, 18, 26, 31, 32, 33, 35, 39, 40 ,42, 43, 44, 45, 51, 52, 53, 56, 58, 59, 66, 68, 69, 70, 72, 73, 81, 84, and 87 | Normal, ASCH, ASCUS, LSIL,HSIL, SCC, and AC |
| Lee et al., 2011^21^ | KoreaMED | Case control study | 342 | 361 | 16-87(45.8) | Reverse blot hybridization assay (REBA) | 6, 11, 16, 18, 31, 33, 35, 39, 42, 43, 44, 45, 51, 52, 53, 56, 58, 59, 68, 70, 72, 84, 81, 87 | Normal, ASCUS, ASCH, LSIL, HSIL, and SCC |
| Kyeong et al., 2009^22^ | KoreaMED | Case control study | 534 | 318 | 15-81 (median; 46) | My Gene Assay  (Mygene) | 6, 11,16, 18, 31, 33, 34, 35, 39, 40, 42, 43, 44, 45,51, 52, 53, 54, 56, 58, 59, 66, 68, and 70 | ASCUS |
| Park et al., 2003^19^ | KoreaMED | Case control study | 394 | 333 | 22-84(mean;46.2) | HPV DNA Chip  (Biomedlab) | 6, 11, 16, 18, 31, 33, 34, 35, 39, 40, 42, 43, 44, 45, 51, 52, 56, 58, 59, 66, 68, and 69 | Normal, ASCUS, AGUS, LSIL, HSIL, and CC |
| Kwon et al., 2004^24^ | KoreaMED | Case control study | 334 | 105 |  | Oligo-DNA Chip | 11, 16, 18, 26, 32, 33, 35, 39, 51, 52, 53, 56, 58, 59, 61, 66, 68, 70, and 72 | Normal, ASCUS, LSIL, and HSIL |
| Lee et al., 2005^39^ | KoreaMED | Case control study | 131 | 75 | 24-66(mean39.65) | My HPV DNA chip  (Mygene) | 16, 58, 56, 18, 39, 52, 66, 33, 35, 31, 53, 54, 59, and LR-HPV | ASCUS |
| Lee et al., 2015^28^ | KMBASE | Cohort study | 144 | 178 | 21-72(median 45) | GG HPV-40 DNA Genotyping Chip (Goodgene) | 16, 18, 26, 31, 33, 35, 39, 45, 51, 52, 53, 56, 58, 59, 66, 67, 68, 69, 70, 73, 82, and Nineteen non-HR HPV types | ASCUS |
| Chung et al., 2013^35^ | KMBASE | Case report | 518 | 61 | 20-89 | HPV genotyping chip  (Goodgene) | 6, 11, 16, 18, 31, 33, 34, 35, 39, 40, 42, 43, 44, 45, 51, 52, 56, 58, 59, 66, 68, 69 and other | Normal, ASCUS, LSIL, and HSIL |
| Ouh et al., 2018^12^ | PubMED | Case control study | 18170 | 2832 | 25-75 (mean;41.4) | HPV Liquid Bead Microarray & nyplex™ II HPV 28(seegene) | 6, 11, 16, 18, 26, 31, 32, 33, 34, 35, 39, 40, 42, 43, 44, 45, 51, 52, 53, 54, 55, 56, 58, 59, 61, 62, 66, 68, 69, 70, 73, 81, and 83 | Normal, ASCUS, and LSIL |
| Cho et al., 2011^20^ | PubMED | Case control study | 624 | 182 |  | Hybrid Capture II assay  (Digene) | 6, 11, 16, 18, 39, 40, 42, 44, 51, 52, 53, 56, 58, 70, and other | Normal, ASCUS, LSIL, HSIL, and SCC |
| Hwang et al., 2004^23^ | PubMED | Case control study | 2,470 | 512 |  | HPV DNA Chip (Biomedlab) | 16,51,52,58, HR-HPV, LR-HPV, and other | Normal, ASCUS, LSIL, and HSIL |
| Park et al., 2004^32^ | PubMED | Case control study | 471 | 296 | 23-68(mean 39.1) | HPV DNA chip  (Biomedlab) | 11, 16, 18, 31, 33, 34, 35, 39, 40, 43, 44, 45, 51, 52, 56, 58, 59, 68, and 69 | Normal, LSIL, HSIL, SC, and AC |
| Seo et al., 2016^33^ | PubMED | Case control study | 470 | 289 | 17-77 (mean 39.1±9.6 | Cheil HPV DNA Chip | 16, 18, 30, 31, 33, 35, 39, 40, 42, 43, 44, 45, 51, 52, 53, 54, 55, 56, 58, 59, 62, 66, 67, 68, 69, 70, 72, 81, 82, 84, 90, 91, and others | Normal, ASCUS/ASC-H, LSIL, HSIL, and CC |
| Tong et al., 2007^38^ | PubMED | Case control study | 97 | 88 | 25-79 (mean 50.34±11.4 | MY HPV DNA chip (Mygene) | HPV-16-related group (type 16, or mixed with other  types, except types 18, 45, and 59), HPV-18-related  group (type 18, 45, 59, or mixed with other types), and  the intermediate-risk group (HPVs 31, 33, 35, | HSIL, ACC, SCC, and AC |
| An et al., 2002^40^ | PubMED | Case control study | 1650 | 549 | Mean 42.34 ±12.03 | HPV DNA chip  (Biomedlab) | 6, 1618, 18, 35, 52, 56, 58, other HR-HPV, LR-HPV, and other type | Normal, ASCUS, AGUS, LSIL, HSIL, and CA |
| Park et al., 2015^29^ | NDSL | Case control study | 4292 | 433 | 17-95 (mean 47.6) | My HPV DNA  Chip  (MyGene) | 6, 11,16, 18, 31, 33, 34, 35, 39, 40, 42, 43, 44, 45, 51, 52, 53, 54, 56, 58, 59, 66, 68, and 70 56, 58, 59, 66, 68, 7 | LSIL, HSIL, and SCC |

Abbreviations: HPV: human papillomavirus; HR-HPV: high-risk human papillomavirus; LR-HPV: low-risk human papillomavirus; ASCUS: atypical squamous cells of undetermined significance; AGUS: atypical glandular cells of undetermined significance; LSIL: low-grade squamous intraepithelial lesion; ASC-H: atypical squamous cells without excluding HSIL; HSIL: high-grade intraepithelial lesion; CIS: carcinoma in situ; ICC: invasive cervical cancer; SCC: squamous cell carcinoma; AC: adenocarcinoma; AIS+: adenocarcinoma in situ and more; AGC: atypical glandular cells; SCCA: squamous cell carcinoma; CC: cervical cancer; AGUS: atypical glandular cells of undetermined significance.
