# Supplementary material for: Enhanced disease progression due to persistent HPV-16/58 infections in Korean women: a systematic review and the Korea HPV cohort study
Source: Virol J. 2021 Sep 17;18:188. doi: 10.1186/s12985-021-01657-2 (PMC8447749; doi:10.1186/s12985-021-01657-2)
Supplement: Supplementary file 2 — Additional file 2. Basic characteristics of women at baseline in the Korea HPV Cohort Study (n = 1,664). [file 12985_2021_1657_MOESM2_ESM.docx]

**Additional Table 2. Basic characteristics of women at baseline in the Korea HPV Cohort Study (n = 1664).**

| Category^*^ | Overall | | ASCUS | | LSIL | | p-value |
| --- | --- | --- | --- | --- | --- | --- | --- |
|  |  |  | n | (%) | n | (%) |  |
| Total | 1664 | | 960 | (57.7) | 704 | (42.3) |  |
| Age (years) |  | |  |  |  |  | 0.001 |
| 20-29 | 319 | | 156 | (48.9) | 163 | (51.1) |  |
| 30-39 | 498 | | 289 | (58.0) | 209 | (42.0) |  |
| 40-49 | 498 | | 299 | (60.0) | 199 | (40.0) |  |
| 50-59 | 317 | | 195 | (61.5) | 122 | (38.5) |  |
| Marital status |  | |  |  |  |  | 0.051 |
| Un-married | 1158 | | 686 | (59.2) | 472 | (40.8) |  |
| Married | 492 | | 266 | (54.1) | 226 | (45.9) |  |
| Education |  | |  |  |  |  | 0.023 |
| ≤ Middle school | 158 | | 102 | (64.6) | 56 | (35.4) |  |
| High school | 470 | | 287 | (61.1) | 183 | (38.9) |  |
| University | 886 | | 482 | (54.4) | 404 | (45.6) |  |
| ≥ Graduate school | 135 | | 81 | (60.0) | 54 | (40.0) |  |
| Job |  | |  |  |  |  | 0.043 |
| Administrator, expert | 384 | | 217 | (56.5) | 167 | (43.5) |  |
| Homemaker | 491 | | 297 | (60.5) | 194 | (39.5) |  |
| Office worker | 318 | | 168 | (52.8) | 150 | (47.2) |  |
| Service, sales | 284 | | 179 | (63.0) | 105 | (37.0) |  |
| Other | 172 | | 91 | (52.9) | 81 | (47.1) |  |
| Family income, KRW^†^ |  | |  |  |  |  | 0.893 |
| ≤ 2000000 | 199 | | 114 | (57.3) | 85 | (42.7) |  |
| 2000000-4000000 | 504 | | 293 | (58.1) | 211 | (41.9) |  |
| 4000000-6000000 | 413 | | 243 | (58.8) | 170 | (41.2) |  |
| 6000000-8000000 | 178 | | 99 | (55.6) | 79 | (44.4) |  |
| > 8000000 | 240 | | 145 | (60.4) | 95 | (39.6) |  |
| Smoking history |  | |  |  |  |  | 0.105 |
| No | 1399 | | 796 | (56.9) | 603 | (43.1) |  |
| Yes | 250 | | 156 | (62.4) | 94 | (37.6) |  |
| Alcohol history |  | |  |  |  |  | 0.010 |
| No | 438 | | 265 | (60.5) | 173 | (39.5) |  |
| Yes | 1028 | | 546 | (53.1) | 482 | (46.9) |  |
| Pregnancy history |  | |  |  |  |  | 0.325 |
| No | 443 | | 247 | (55.8) | 196 | (44.2) |  |
| Yes | 1206 | | 705 | (58.5) | 501 | (41.5) |  |
| Oral contraceptive pill history |  | |  |  |  |  | 0.150 |
| No | 1342 | | 786 | (58.6) | 556 | (41.4) |  |
| Yes | 307 | | 166 | (54.1) | 141 | (45.9) |  |
| Age of sexual debut |  | |  |  |  |  | 0.007 |
| 13-19 | 236 | | 131 | (55.5) | 105 | (44.5) |  |
| 20-24 | 755 | | 467 | (61.9) | 288 | (38.1) |  |
| >25 | 654 | | 352 | (53.8) | 302 | (46.2) |  |
| No. of sex partners in lifetime |  | |  |  |  |  | 0.498 |
| 1 | 585 | | 336 | (57.4) | 249 | (42.6) |  |
| 2-3 | 601 | | 343 | (57.1) | 258 | (42.9) |  |
| 4-5 | 272 | | 160 | (58.8) | 112 | (41.2) |  |
| > 6 | 131 | | 84 | (64.1) | 47 | (35.9) |  |
| Family history of cervical cancer | |  |  |  |  |  | 0.940 |
| Yes | 93 | | 54 | (58.1) | 39 | (41.9) |  |
| No | 1571 | | 906 | (57.7) | 665 | (42.3) |  |
| Baseline HPV genotype^‡^ |  | |  |  |  |  |  |
| High-risk | 1744 | | 1005 | (57.6) | 739 | (42.4) | 0.001 |
| HPV-16 | 204 | | 129 | (63.2) | 75 | (36.8) |  |
| HPV-18 | 94 | | 60 | (63.8) | 34 | (36.2) |  |
| HPV-26 | 3 | | 1 | (33.3) | 2 | (66.7) |  |
| HPV-31 | 49 | | 32 | (65.3) | 17 | (34.7) |  |
| HPV-33 | 52 | | 29 | (55.8) | 23 | (44.2) |  |
| HPV-35 | 66 | | 40 | (60.6) | 26 | (39.4) |  |
| HPV-39 | 123 | | 79 | (64.2) | 44 | (35.8) |  |
| HPV-45 | 30 | | 18 | (60.0) | 12 | (40.0) |  |
| HPV-51 | 128 | | 66 | (51.6) | 62 | (48.4) |  |
| HPV-52 | 173 | | 115 | (66.5) | 58 | (33.5) |  |
| HPV-53 | 173 | | 85 | (49.1) | 88 | (50.9) |  |
| HPV-56 | 177 | | 77 | (43.5) | 100 | (56.5) |  |
| HPV-58 | 180 | | 105 | (58.3) | 75 | (41.7) |  |
| HPV-59 | 49 | | 33 | (67.4) | 16 | (32.6) |  |
| HPV-66 | 99 | | 39 | (39.4) | 60 | (60.6) |  |
| HPV-68 | 119 | | 81 | (68.1) | 38 | (31.9) |  |
| HPV-73 | 5 | | 2 | (40.0) | 3 | (60.0) |  |
| HPV-82 | 20 | | 14 | (70.0) | 6 | (30.0) |  |
| Low-risk | 455 | | 263 | (57.8) | 192 | (42.2) | 0.309 |
| HPV-6 | 39 | | 23 | (59.0) | 16 | (41.0) |  |
| HPV-11 | 19 | | 9 | (47.4) | 10 | (52.6) |  |
| HPV-40 | 53 | | 27 | (50.9) | 26 | (49.1) |  |
| HPV-42 | 47 | | 29 | (61.7) | 18 | (38.3) |  |
| HPV-43 | 35 | | 22 | (62.9) | 13 | (37.1) |  |
| HPV-44 | 49 | | 27 | (55.1) | 22 | (44.9) |  |
| HPV-54 | 71 | | 36 | (50.7) | 35 | (49.3) |  |
| HPV-61 | 30 | | 22 | (73.3) | 8 | (26.7) |  |
| HPV-70 | 82 | | 53 | (64.6) | 29 | (35.4) |  |
| HPV-72 | 2 | | 2 | (100) | 0 | (0.0) |  |
| HPV-81 | 28 | | 13 | (46.4) | 15 | (53.6) |  |

Abbreviations: ASCUS: atypical squamous cells of undetermined significance; LSIL: low-grade squamous intraepithelial lesion; HPV human papilloma virus.

^*^Missing values were excluded from the analysis. ^†^The currency exchange rate: 1 U.S Dollar (USD) was approximately 1208 KRW (1,000,000 KRW = 824.47 USD). ^‡^The frequencies of each HPV type when it was found in each sample.
