# Supplementary material for: Enhanced disease progression due to persistent HPV-16/58 infections in Korean women: a systematic review and the Korea HPV cohort study
Source: Virol J. 2021 Sep 17;18:188. doi: 10.1186/s12985-021-01657-2 (PMC8447749; doi:10.1186/s12985-021-01657-2)
Supplement: Supplementary file 3 — Additional file 3. Risk of disease progression to HSIL by persistent HPV infections (single/multi-infection). [file 12985_2021_1657_MOESM3_ESM.docx]

**Additional Table 3. Risk of disease progression to HSIL by persistent HPV infections (single/multi-infection).**

|  | N | Progression | Hazard ratio (95% CI) | | P-value^†^ |
| --- | --- | --- | --- | --- | --- |
|  |  |  | Unadjusted | Adjusted^*^ |  |
| Single persistent infection | 68 | 20 | Ref | Ref |  |
| Multiple persistent infections | 45 | 8 | 0.76 (0.33-1.73) | 0.99 (0.40-2.47) | 0.986 |

Abbreviations: HSIL: High-grade squamous intraepithelial lesion; HPV: Human Papillomavirus; 95% CI: 95% confidence interval.

^*^Hazard ratios and 95% confidence intervals were adjusted by age. ^†^P-values were calculated in the adjusted analysis.
